# Risk of extended major adverse cardiovascular event endpoints with tofacitinib versus TNF inhibitors in patients with rheumatoid arthritis: a post hoc analysis of a phase 3b/4 randomised safety study

**DOI:** 10.1136/rmdopen-2023-003912

**Published:** 2024-04-12

**Authors:** Maya H Buch, Deepak L Bhatt, Christina Charles-Schoeman, Jon T Giles, Ted Mikuls, Gary G Koch, Steven Ytterberg, Edward Nagy, Hyejin Jo, Kenneth Kwok, Carol A Connell, Karim Richard Masri, Arne Yndestad

**Affiliations:** 1 Centre for Musculoskeletal Research, Division of Musculoskeletal and Dermatological Sciences, The University of Manchester, Manchester, UK; 2 NIHR Manchester Biomedical Research Centre, Manchester, UK; 3 Mount Sinai Heart, Icahn School of Medicine at Mount Sinai Health System, New York, New York, USA; 4 Division of Rheumatology, Department of Medicine, University of California, Los Angeles, California, USA; 5 Division of Rheumatology, Columbia University, College of Physicians and Surgeons, New York, New York, USA; 6 Department of Medicine, University of Nebraska Medical Center, Omaha, Nebraska, USA; 7 University of North Carolina at Chapel Hill Department of Biostatistics, Chapel Hill, North Carolina, USA; 8 Division of Rheumatology, Mayo Clinic, Rochester, Minnesota, USA; 9 Pfizer Ltd, Tadworth, UK; 10 Pfizer Inc, New York, New York, USA; 11 Pfizer Inc, Groton, Connecticut, USA; 12 Pfizer Inc, Collegeville, Pennsylvania, USA; 13 Pfizer Inc, Oslo, Norway

**Keywords:** Antirheumatic Agents, Cardiovascular Diseases, Therapeutics, Arthritis, Rheumatoid, Tumor Necrosis Factor Inhibitors

## Abstract

**Objectives:**

Compare the risk of extended major adverse cardiovascular (CV) event (MACE) composite outcomes and component events in patients with rheumatoid arthritis (RA) treated with tofacitinib versus tumour necrosis factor inhibitors (TNFi) in Oral Rheumatoid Arthritis Trial (ORAL) Surveillance.

**Methods:**

Patients with RA aged ≥50 years and with ≥1 additional CV risk factor received tofacitinib 5 mg or 10 mg two times per day or TNFi. MACE (non-fatal myocardial infarction (MI), non-fatal stroke or CV death (MACE-3)) was extended by sequential addition of CV events (hospitalisation for unstable angina (MACE-4), coronary revascularisation (MACE-5), transient ischaemic attack (MACE-6), peripheral vascular disease (MACE-7)), heart failure (HF) hospitalisation (MACE-8) and venous thromboembolism (VTE; (MACE-8 plus VTE)). HRs (tofacitinib vs TNFi) were evaluated for MACE and individual components.

**Results:**

HRs for MACE-4 to MACE-8 with combined and individual tofacitinib doses versus TNFi were similar. Risk of MACE-8 plus VTE appeared similar with tofacitinib 5 mg two times per day versus TNFi (HR 1.12 (0.82 to 1.52)), but higher with tofacitinib 10 mg two times per day versus TNFi (HR 1.38 (1.02 to 1.85)). Risk of MI was higher with tofacitinib versus TNFi, but difference in risk of other individual CV events was not suggested. Across extended MACE definitions, risk appeared higher with tofacitinib versus TNFi in those with atherosclerotic CV disease or age ≥65 years.

**Conclusion:**

In ORAL Surveillance, risk of composite CV endpoints combining all ischaemic CV events and HF did not appear different with tofacitinib versus TNFi. The totality of CV risk was higher with tofacitinib 10 mg two times per day versus TNFi, driven by an increase in VTE.

**Trial registration number:**

NCT02092467.

**WHAT IS ALREADY KNOWN ON THIS TOPIC**

- ORAL Surveillance indicated that patients with RA aged ≥50 years with ≥1 additional cardiovascular (CV) risk factor have an increased risk of major adverse CV events (MACE) and malignancies (excluding non-melanoma skin cancer (NMSC)) with tofacitinib compared with tumour necrosis factor inhibitors (TNFi).
- A difference in risk of MACE, myocardial infarction (MI), venous thromboembolism (VTE), malignancies (excluding NMSC) and all-cause death with tofacitinib versus TNFi was not apparent in patients who were <65 years of age that had never smoked.
- Increased risk of MACE with tofacitinib versus TNFi was mainly apparent in patients with a history of atherosclerotic CV disease (ASCVD).

**WHAT THIS STUDY ADDS**

- This post hoc analysis of ORAL Surveillance shows a similar risk of a composite of all ischaemic CV events (MACE-7) with tofacitinib versus TNFi, that is, MI and VTE are the key CV outcomes increased with tofacitinib versus TNFi.
- The totality of CV risk, that is, composite of all adjudicated CV events including VTE (MACE-8 plus VTE) was similar with tofacitinib 5 mg two times per day vs TNFi. But it was higher with tofacitinib 10 mg two times per day vs TNFi, driven by an increase in VTE events.
- Across extended composites of MACE, risk appeared higher with tofacitinib vs TNFi in patients with a history of ASCVD and in patients who were ≥65 years of age, but similar among risk categories in patients with no history of ASCVD and in patients <65 years of age.

**HOW THIS STUDY MIGHT AFFECT RESEARCH, PRACTICE OR POLICY**

- Our analysis provides clinicians with a better appreciation of the totality of CV risk associated with tofacitinib versus TNFi and inform further on the importance of higher age and history of ASCVD in the individualised risk assessment when considering treatment with tofacitinib relative to TNFi.
- These findings highlight the need for greater understanding of overall CV risk and risk of individual CV events in patients with RA.

## Introduction

People with rheumatoid arthritis (RA) are at increased risk of a spectrum of cardiovascular diseases (CVD), including atherosclerotic CVD (ASCVD), venous thromboembolism (VTE), atrial fibrillation and heart failure (HF).^1–8^ ASCVD is the most common form of CVD in people with RA and the general population,^2^ and depending on the vascular bed affected, it can result in acute coronary syndromes such as myocardial infarction (MI) and unstable angina pectoris (UAP), cerebrovascular events such as ischaemic stroke and transient ischaemic attack (TIA) and peripheral vascular disease (PVD), such as peripheral artery occlusion.^9^

Oral Rheumatoid Arthritis Trial ORAL Surveillance was a United States Food and Drug Administration required postauthorisation safety study in patients with RA aged ≥50 years with at least one additional CV risk factor and was designed and powered to assess non-inferiority of combined tofacitinib doses (5 mg and 10 mg two times per day) vs TNF inhibitors (TNFi) for the coprimary endpoints of adjudicated major adverse CV events (MACE) and malignancy (excluding non-melanoma skin cancer (NMSC)).^10^ Tofacitinib non-inferiority would be shown if the upper limit of the two-sided 95% CI for the HR was less than 1.8 for the combined tofacitinib doses versus TNFi.^10^ Importantly, this non-inferiority criterion did not apply to comparison of the individual tofacitinib doses versus TNFi or for the assessment of other safety outcomes. The study did not demonstrate non-inferiority of tofacitinib versus TNFi for MACE and malignancy (excluding NMSC).^10 11^

MACE was in ORAL Surveillance defined as the composite of adjudicated CV death, non-fatal MI and non-fatal stroke (ie, three-point MACE; hereafter referred to as MACE-3).^10^ Risk of MACE-3 and MI was higher with tofacitinib 5 mg and 10 mg two times per day versus TNFi, and risk of VTE was higher with tofacitinib 10 mg two times per day vs TNFi.^10 12^ A post hoc analysis of ORAL Surveillance found higher risk of MACE-3 with tofacitinib versus TNFi in patients with a history of ASCVD. However, risk did not differ with tofacitinib 5 mg two times per day versus TNFi in patients without a history of ASCVD.^12^

Extended versions of MACE-3 including one or more additional adjudicated CV events in the composite endpoint provide a wider assessment of type of CV risk associated with a certain treatment. The totality of CV risk can be evaluated in composites that include CV events regardless of pathogenic processes, for example, by combining ischaemic CV events, HF, and VTE. By including extended MACE endpoints, more events are also captured and a better precision in the risk assessment can potentially be achieved.

This post hoc analysis aimed to expand on the primary and secondary analyses of ORAL Surveillance by (1) addressing the totality of CV risk with tofacitinib vs TNFi by evaluation of all adjudicated CV events as part of extended MACE endpoints, (2) assessing whether the identified risk of MI with tofacitinib versus TNFi demonstrated in ORAL Surveillance also applied to other CV events^10^ and (3) applying the previously reported analyses by history of ASCVD and by age across extended MACE endpoints.^12^

## Methods

### Study design and patients

ORAL Surveillance was a phase 3b/4 randomised, open-label, non-inferiority, safety endpoint study conducted from March 2014–July 2020 in patients with RA. Eligibility comprised active moderate-to-severe RA despite methotrexate treatment, age ≥50 years with ≥1 additional CV risk factor (current smoking, hypertension, high-density lipoprotein cholesterol <40 mg/dL, diabetes mellitus, family history of premature coronary heart disease, RA-associated extra articular disease and/or history of CAD).^10^

Participants were randomised 1:1:1 to receive oral tofacitinib 5 or 10 mg two times per day or subcutaneous TNFi (adalimumab 40 mg every 2 weeks (North America; ie, USA, Puerto Rico and Canada) or etanercept 50 mg once weekly (rest of the world)). All patients continued their prestudy stable dose of methotrexate unless modification was clinically indicated. In February 2019, the tofacitinib 10 mg two times per day dose was reduced to 5 mg two times per day after the Data Safety Monitoring Board noted an increased frequency of pulmonary embolism (PE) in patients receiving tofacitinib 10 mg two times per day versus TNFi and an increase in overall mortality with tofacitinib 10 versus 5 mg two times per day and TNFi.

ORAL Surveillance was conducted in accordance with the Declaration of Helsinki and Good Clinical Practice Guidelines of the International Council on Harmonisation, and local country regulations, and was approved by the Institutional Review Board and/or Independent Ethics Committee at each centre. Patients provided written informed consent.

### CV outcomes

In ORAL Surveillance, MACE-3 was defined as the composite of CV death (ie, death due to MI, stroke, sudden cardiac death, HF, CV procedures, CV haemorrhage and other CV causes, but not death due to PE), non-fatal MI and non-fatal stroke (including reversible focal neurological defects with imaging evidence of a new cerebral lesion consistent with ischaemia or haemorrhage) (table 1). These events were adjudicated by an external, independent committee. Other adjudicated CV events not accounted for in the MACE-3 composite were hospitalisation for UAP, coronary revascularisation, TIA, PVD, hospitalisation for HF and VTE (deep vein thrombosis and/or PE). As shown in table 1, extended MACE endpoints to MACE-3 comprised sequential addition of adjudicated ischaemic CV events to the composite (ie, UAP (MACE-4), coronary revascularisation (MACE-5), TIA (MACE-6) and PVD [MACE-7; all ischaemic CV events)), HF (MACE-8) and VTE (MACE-8 plus VTE). Outcome was met at the first occurrence of one of the CV events included in the composite definition (table 1).

**Table 1:** Definition of extended MACE endpoints

CV event	MACE-3*	MACE-4	MACE-5	MACE-6	MACE-7†	MACE-8	MACE-8 plus VTE
CV death	X	X	X	X	X	X	X
Myocardial infarction (non-fatal)	X	X	X	X	X	X	X
Stroke (non-fatal)	X	X	X	X	X	X	X
Hospitalisation for unstable angina (UAP)		X	X	X	X	X	X
Coronary revascularisation			X	X	X	X	X
Transient ischaemic attack (TIA)				X	X	X	X
Peripheral vascular disease (PVD)					X	X	X
Hospitalisation for heart failure (HF)						X	X
Venous thromboembolism (VTE)							X

The numeral after MACE refers to the number of adjudicated CV events included in the composite. The specific CV events (first column) are only included in the MACE composites indicated with ‘X’ in the respective row.

*Coprimary endpoint in ORAL Surveillance.

†All ischaemic CV events.

CV, cardiovascular; MACE, major adverse CV event; ORAL, Oral Rheumatoid Arthritis Trial.

Two outcomes were defined for adjudicated events of coronary revascularisation (either percutaneous coronary intervention (PCI) or coronary artery bypass grafting (CABG)). Total coronary revascularisation included all events. A separate outcome was defined for coronary revascularisation that was not associated with a concomitant (ie, same day) event of MI or UAP. Events of atrial fibrillation were not adjudicated in ORAL Surveillance and were identified as MedDRA version V.23.1 preferred term ‘atrial fibrillation’.

The safety analysis set used to analyse outcomes included all randomised participants receiving ≥1 dose of study drug. Data collected for patients randomised to tofacitinib 10 mg two times per day who had their dose reduced to 5 mg two times per day in February 2019 were maintained in the tofacitinib 10 mg two times per day group. All adjudicated CV events (including VTE) were counted within the risk period, defined as time from first to last study dose+60 days or to last contact date (ie, if a patient died, last contact date was death date), whichever was earliest. Patients without events were censored at the end of the risk period. For atrial fibrillation, a 28-day on treatment time risk period was applied.

### Statistical analyses

HRs (time to first event analysis) were evaluated with tofacitinib versus TNFi for extended MACE endpoints (risk period up to first event of aggregated CV events) and for individual CV outcomes (risk period up to first event of individual CV outcome), separately. HRs (95% CIs) were estimated based on two simple Cox proportional hazard regression models (one for comparing tofacitinib 5 mg two times per day and 10 mg two times per day each vs TNFi, and the other for comparing combined tofacitinib combined doses vs TNFi) with treatment as the only covariate. The non-inferiority criterion of the primary endpoint analysis did not apply, and results were assessed based on the HR point estimate and the 95% CI. Crude, exposure-adjusted incidence rates were expressed as the number of patients with first event per 100 patient-years, along with two-sided 95% CIs using the exact Poisson method. Cumulative probability plots using Kaplan-Meier estimates were generated for analysis of time to event for MACE-7, MACE-8, and MACE-8 plus VTE.

For MACE-7 (all ischaemic CV events), an analysis was performed that also included recurrent/subsequent events. Multiple events occurring on same day were considered as a single event. Risk of total ischaemic CV events (HR (95% CI)) with tofacitinib versus TNFi was estimated using the Anderson-Gill model.^13^

For all outcomes, subgroup analyses were performed by history of ASCVD, by age (≥65 or <65 years) at the time of study enrolment, and by geographical region (North America or rest of the world). As previously described,^12^ in patients without a history of ASCVD, 10-year risk of events associated with ASCVD (ie, MACE) was calculated by ASCVD-Pooled Cohort Equations (ASCVD-PCE), applying a 1.5 multiplier per EULAR recommendations.^3 14^

All analyses were post hoc. Across these exploratory analyses, no multiplicity adjustments were applied.

## Results

### Study population and baseline characteristics

Patient demographics and baseline disease characteristics are described elsewhere.^10 12^ Table 2 summarises CV risk factors and CV risk profile of the study population. These were balanced across the treatment arms. Approximately 15% of patients had a history of ASCVD, and of patients with no history of ASCVD, approximately 50% had intermediate-high (ie, ≥7.5% 10-year risk of MACE). Online supplemental tables 1 and 2 summarises characteristics of patients by age (≥65 or <65 years) and geographical region (North America (USA, Puerto Rico and Canada) or rest of the world).

10.1136/rmdopen-2023-003912.supp1 Supplementary data

**Table 2:** Key demographic and baseline disease characteristics of patients in ORAL Surveillance

	Tofacitinib 5 mg two times per day (N=1455)	Tofacitinib 10 mg two times per day (N=1456)	TNFi (N=1451)
Age (years), mean (SD)	60.8 (6.8)	61.4 (7.1)	61.3 (7.5)
≥65 years, n (%)	413 (28.4%)	478 (32.8%)	462 (31.8%)
Female sex, n (%)	1169 (80.3%)	1124 (77.2%)	1117 (77.0%)
Race*, n (%)			
White	1128 (77.5%)	1126 (77.3%)	1099 (75.7%)
Black	63 (4.3%)	65 (4.5%)	83 (5.7%)
Asian	65 (4.5%)	56 (3.8%)	55 (3.8%)
Other	199 (13.7%)	209 (14.4%)	214 (14.7%)
Smoking status, n (%)			
Current smoker	411 (28.2%)	402 (27.6%)	353 (24.3%)
Past smoker	309 (21.2%)	302 (20.7%)	326 (22.5%)
Never smoked	735 (50.5%)	752 (51.6%)	772 (53.2%)
Smoking duration, years (SD)†			
Current smokers	32.2 (13.1)	33.0 (13.0)	31.9 (13.2)
Past smokers	36.5 (13.0)	38.1 (13.0)	39.9 (12.2)
Time since smoking cessation (past smokers), years (SD)	14.6 (12.2)	16.3 (12.0)	18.8 (13.1)
BMI (kg/m^2^) ≥30‡, n (%)	606 (41.6%)	594 (40.8%)	617 (42.5%)
History of diabetes mellitus, n (%)	243 (16.7%)	261 (17.9%)	255 (17.6%)
History of hypertension, n (%)	955 (65.6%)	954 (65.5%)	969 (66.8%)
History of hyperlipidaemia, n (%)	525 (36.1%)	518 (35.6%)	491 (33.8%)
Baseline statins,§ n (%)	394 (24.0%)	350 (24.0%)	321 (22.1%)
Baseline aspirin,^§^ n (%)	226 (15.5%)	244 (16.8%)	237 (16.3%)
History of ASCVD, n (%)	204 (14.0%)	222 (15.2%)	214 (14.7%)
10-year risk of MACE¶ n (%)			
High (≥20%)	258 (17.7%)	289 (19.8%)	278 (19.2%)
Intermediate (≥7.5–<20%)	472 (32.4%)	490 (33.7%)	483 (33.3%)
Borderline (≥5–<7.5%)	198 (13.6%)	169 (11.6%)	153 (10.5%)
Low (<5%)	306 (21.0%)	268 (18.4%)	308 (21.2%)

*Race was reported by the patient.

†Information on smoking duration was missing on eight patients (two current and six past smokers) treated with tofacitinib and five patients (one current and four past smokers) treated with TNFi. In the past smokers, data were also missing on time since smoking cessation.

‡Across treatment groups, data were missing in 17 patients (0.4%).

§Based on day 1 of treatment with tofacitinib or TNFi in ORAL Surveillance.

¶In patients with no history of ASCVD, a 10-year risk of MACE was calculated with the ASCVD-PCE calculator,^14^ and a 1.5 multiplier was applied for RA, as recommended by EULAR.^3^ Percentages are calculated based on the total N.

ASCVD, atherosclerotic cardiovascular disease; ASCVD-PCE, ASCVD-Pooled Cohort Equations; BMI, Body Mass Index; EULAR, European Alliance of Associations for Rheumatology; MACE, major adverse cardiovascular event; n, number of patients with a characteristic; N, number of evaluable patients; ORAL, Oral Rheumatoid Arthritis Trial; TNFi, tumour necrosis factor inhibitors.

### Risk of ischaemic CV events with tofacitinib versus TNFi in ORAL surveillance

MACE-3 events were recorded in 135 of 4362 participants (3.1%) as reported in ORAL Surveillance.^10^ When extending the MACE-3 composite to include all ischaemic CV outcomes, that is, MACE-7, events occurred in a total of 197 of 4362 patients (4.5%; 66/1455 (4.5%) in the tofacitinib 5 mg two times per day group, 68/1456 (4.7%) in the tofacitinib 10 mg two times per day group, and 63/1451 (4.3%) in the TNFi group.

**Figure 1:**
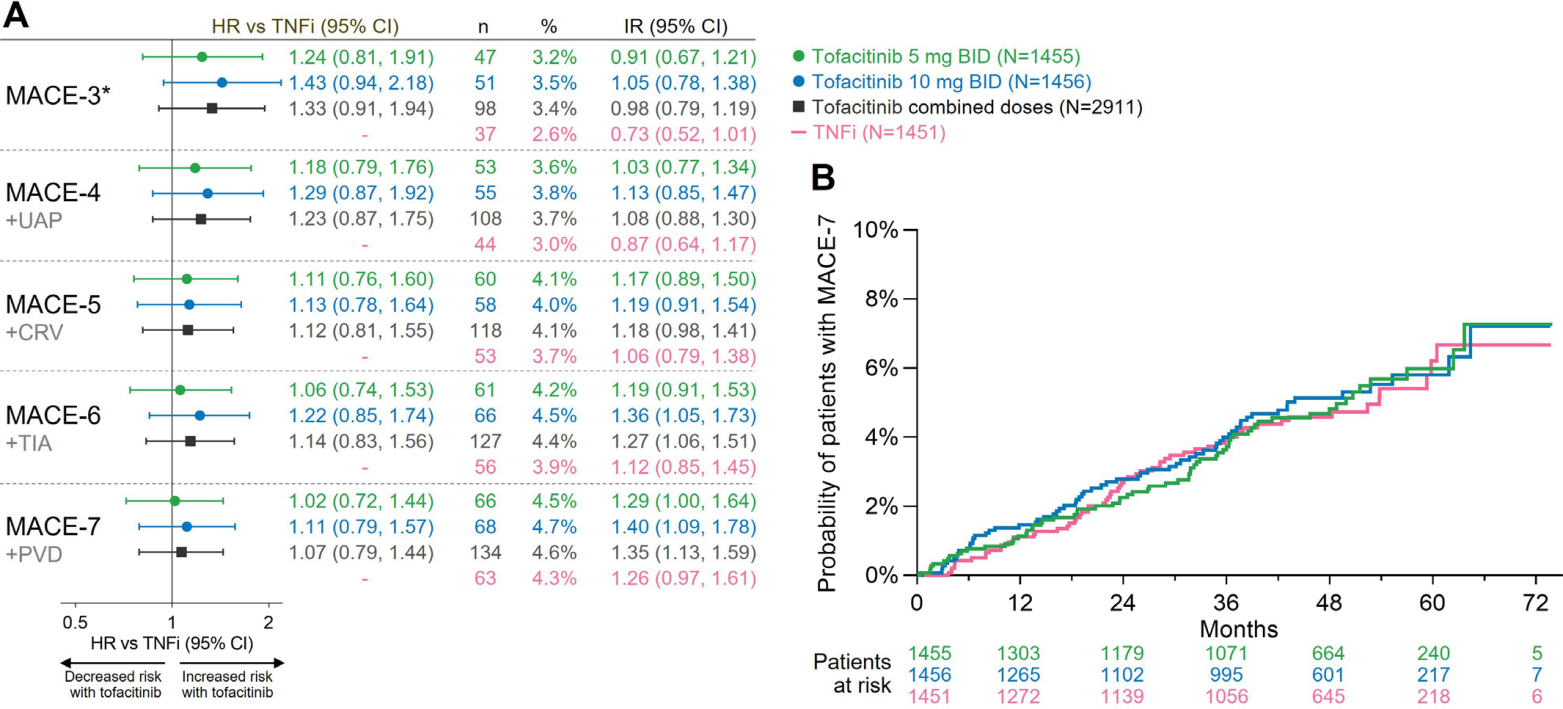
Risk of extended MACE endpoints as composites of ischaemic CV events with tofacitinib versus TNFi. (A) HRs (95% CIs), shown on a logarithmic scale, are from time to first event analyses based on two simple Cox proportional hazard models: one comparing tofacitinib 5 mg two times per day and 10 mg two times per day versus TNFi, and the other comparing combined tofacitinib doses versus TNFi. IRs express the number of patients with first events per 100 PY. *Co-primary endpoint in ORAL Surveillance. Previously reported in Ytterberg *et al*,^10^ and included for reference. (B) Cumulative probability of patients with MACE-7/all ischaemic CV events, calculated based on the Kaplan-Meier estimate. CRV, coronary revascularisation; CV, cardiovascular; IR, incidence rate; MACE, major adverse CV events; N, number of evaluable patients; n, number of patients with events; PVD, peripheral vascular disease; PY, patient-years; TNFi, tumour necrosis factor inhibitor; UAP, hospitalisation for unstable angina pectoris.

For the extended composite endpoints that included ischaemic CV events (ie, MACE-4 to MACE-7), HRs with 95% CI for tofacitinib versus TNFi for time-to-first event were similar to those previously reported for MACE-3, the coprimary endpoint in ORAL Surveillance (figure 1A).^10^ As MACE-3 was sequentially extended to include other CV and vascular events, the HR estimates moved closer to 1.0 and the 95% CI were narrower. For the composite of all ischaemic CV events, MACE-7, the HR with combined tofacitinib doses versus TNFi was 1.07 (0.79, 1.44). Kaplan-Meier curves for MACE-7 did not indicate any separation between the tofacitinib and TNFi groups (figure 1B).

There were few patients with >1 ischaemic CV event during the trial, and the risk of total (ie, first and subsequent) ischaemic CV events for MACE-7 was similar with combined tofacitinib versus TNFi (HR 1.04 (0.78 to 1.38)) (online supplemental figure 1).

### Totality of CV risk with tofacitinib versus TNFi

**Figure 2:**
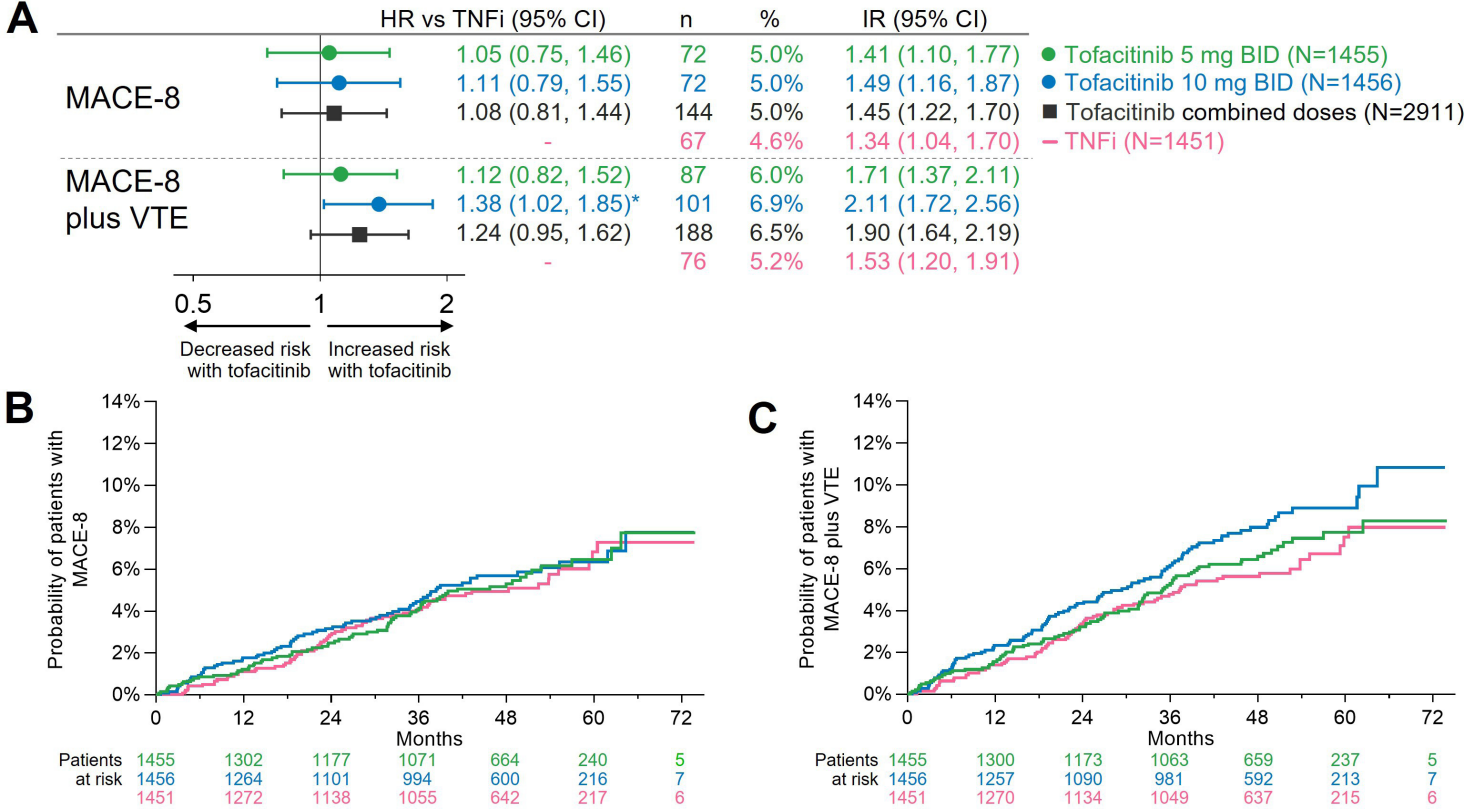
Totality of cardiovascular risk with tofacitinib versus TNFi as estimated by MACE-8 and MACE-8 plus VTE. (A) HRs (95% CIs), shown on a logarithmic scale, are from time to first event analyses based on two simple Cox proportional hazard models: one comparing tofacitinib 5 mg two times per day and 10 mg two times per day versus TNFi, and the other comparing combined tofacitinib doses versus TNFi. IRs express the number of patients with first events per 100 PY. *HR 95% CI excludes 1. Cumulative probabilities of (B) patients with MACE-8 and (C) patients with MACE-8 plus VTE, calculated based on the Kaplan-Meier estimates. ‘MACE-8’ and ‘MACE-8 plus VTE’ were composites of all adjudicated CV events in ORAL Surveillance, without and with the inclusion of VTE, respectively. CV, cardiovascular; IR, incidence rate; MACE, major adverse cardiovascular events; N, number of evaluable patients; n, number of patients with events; PY, patient-years; TNFi, tumour necrosis factor inhibitor; VTE, venous thromboembolism.

The totality of CV risk with tofacitinib versus TNFi was assessed with the endpoints MACE-8 (all ischaemic events plus HF hospitalisation) and MACE-8 plus VTE. As shown in figure 2A, risk of MACE-8 was similar with tofacitinib versus TNFi (HR for combined tofacitinib doses vs TNFi, 1.08 (0.81 to 1.44)). A difference in risk of MACE-8 plus VTE with tofacitinib 5 mg two times per day vs TNFi (HR 1.12 (0.82 to 1.52)) was not suggested. However, there was an increased risk of MACE-8 plus VTE with tofacitinib 10 mg two times per day versus TNFi (HR 1.38 (1.02 to 1.85)).

The Kaplan-Meier curve for MACE-8 did not indicate any separation between the tofacitinib and TNFi groups. However, with MACE-8 plus VTE, the curve representing tofacitinib 10 mg two times per day separated from the other curves beginning at approximately 6 months (figure 2B).

### Risk of individual adjudicated CV events with tofacitinib versus TNFi

**Figure 3:**
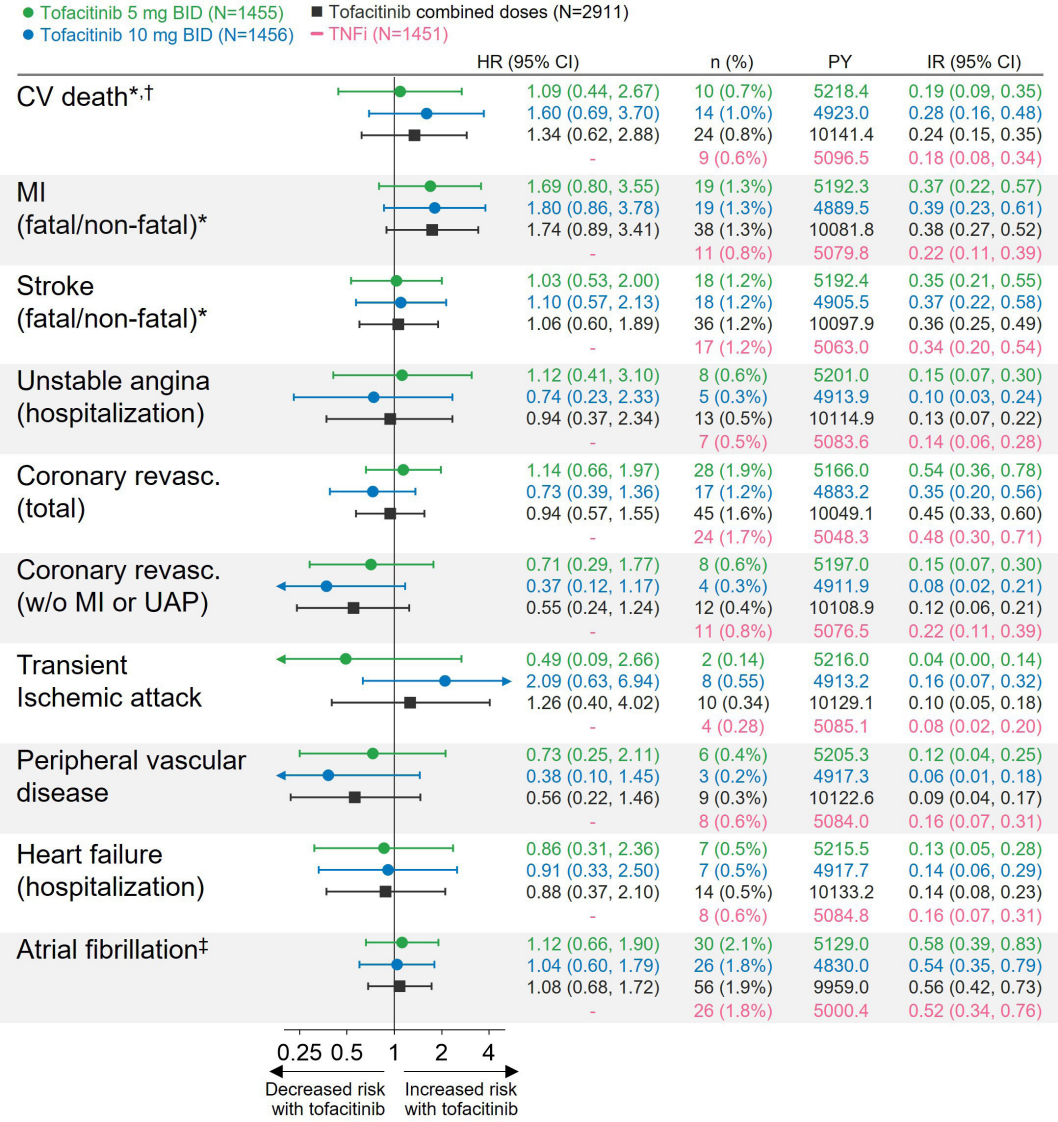
Risk of individual adjudicated CV events with tofacitinib versus TNFi. *Results reported in Charles-Schoeman *et al* 12 and included for reference. ^†^CV death was here defined as death due to sudden cardiac death, fatal heart failure, cardiogenic shock (pump failure), or other vascular death, and did not include fatal MI or stroke. ^‡^Events were not adjudicated and were identified through MedDRA preferred term. HRs (95% CIs), shown on a logarithmic scale, are from time to first event analyses based on two simple Cox proportional hazard models: one comparing tofacitinib 5 mg two times per day and 10 mg two times per day versus TNFi, and the other comparing combined tofacitinib doses versus TNFi. IRs express the number of patients with first events per 100 PY. IR, incidence rate; MACE, major adverse cardiovascular events; MI, myocardial infarction; N, number of evaluable patients; n, number of patients with events; PY, patient-years; revasc., revascularisation; TNFi, tumour necrosis factor inhibitor; UA, unstable angina pectoris.

Figure 3 shows adjudicated CV events in ORAL Surveillance. Across treatment arms, 1.6% (69/4362; 28/1455 (1.9%) with tofacitinib 5 mg two times per day, 17/1456 (1.2%) with tofacitinib 10 mg two times per day and 24/1451 (1.7%) with TNFi) of patients had a coronary revascularisation procedure during the trial, and this was the most frequent adjudicated CV event. Most coronary revascularisation procedures were associated with adjudicated events of MI or hospitalisation for unstable angina (tofacitinib 5 mg two times per day, 71.4% (20/28); tofacitinib 10 mg two times per day, 76.5% (13/17); TNFi 54.2% (13/24)). Risk of coronary revascularisation was similar with tofacitinib versus TNFi (HR 0.94 (0.57 to 1.55)). Notably, risk of coronary revascularisation not associated with MI or UAP was numerically lower with tofacitinib versus TNFi (HR 0.55 (0.24 to 1.24)).

We previously reported on the increased risk of MI with tofacitinib versus TNFi in ORAL Surveillance.^12^ Online supplemental table 3 outlines events of MI per fatal or non-fatal, and by classification as STEMI (ie, MI with ST-elevation on ECG reflective of complete coronary artery occlusion) or NSTEMI (ie, MI without ST-elevation reflective of partial or intermittent occlusion of the coronary artery).^15^ Compared with MI and stroke, other individual ischaemic CV events, ie, UAP, TIA and PVD, were relatively rare (figure 3). There were also few events of hospitalisation for HF. No difference in risk of these events was apparent with tofacitinib versus TNFi, but due to the low number of events, these results should be interpreted with caution.

Adverse events of atrial fibrillation were reported in 2.1% (30/1455) of patients treated with tofacitinib 5 mg two times per day, 1.8% (26/1456) with tofacitinib 10 mg two times per day, and 1.8% (26/1451) with TNFi. Risk of atrial fibrillation was similar with tofacitinib versus TNFi (HR 1.08 (0.68 to 1.72); figure 3).

### Risk of extended MACE endpoints with tofacitinib versus TNFi by history of ASCVD

**Figure 4:**
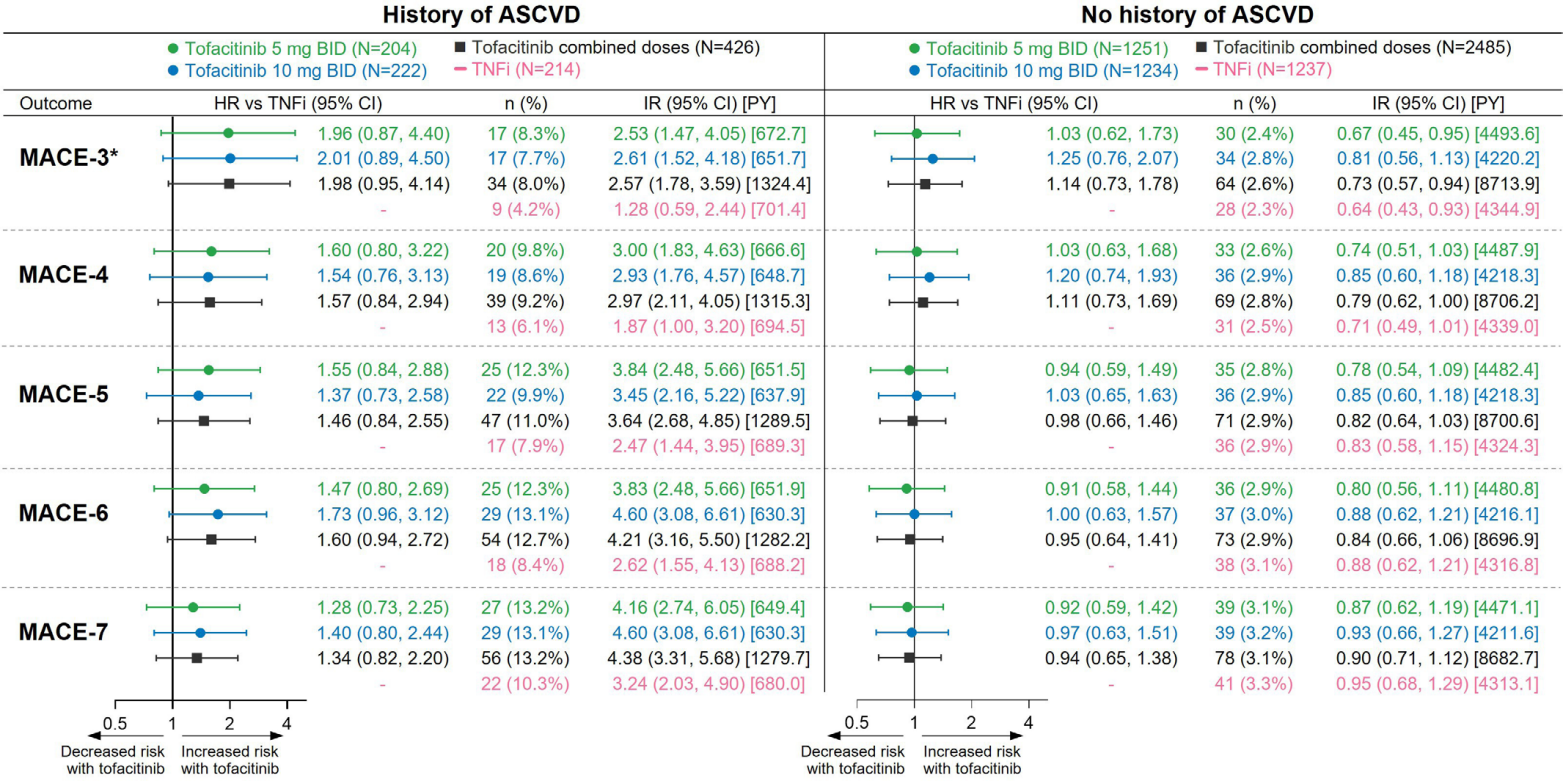
Risk of extended MACE endpoints with tofacitinib versus TNFi in by history of ASCVD.*Results reported in Charles-Schoeman *et al* 12 and included for reference. HRs (95% CIs), shown on a logarithmic scale, are from time to first event analyses based on two simple Cox proportional hazard models: one comparing tofacitinib 5 mg two times per day and 10 mg two times per day versus TNFi, and the other comparing combined tofacitinib doses versus TNFi. IRs express the number of patients with first events per 100 PY. ASCVD, atherosclerotic cardiovascular disease; IR, incidence rate; MACE, major adverse cardiovascular events; N, number of evaluable patients; n, number of patients with events; PY, patient-years; TNFi, tumour necrosis factor inhibitor.

Across treatment groups, the absolute risk of extended MACE endpoints was markedly higher in patients with a history of ASCVD versus those with no history of ASCVD (figure 4). The HR of MACE-8 in patients with history of ASCVD versus no history of ASCVD was 5.30 (3.81 to 7.37) in patients treated with tofacitinib and 3.79 (2.31 to 6.22) in the TNFi group. A previous post hoc analysis showed that increased risk of MACE-3 with tofacitinib versus TNFi was mainly observed in patients with a history of ASCVD.^12^ This pattern was consistent across the extended MACE definitions (ie, MACE-4 to MACE-8) (figure 4).

**Figure 5:**
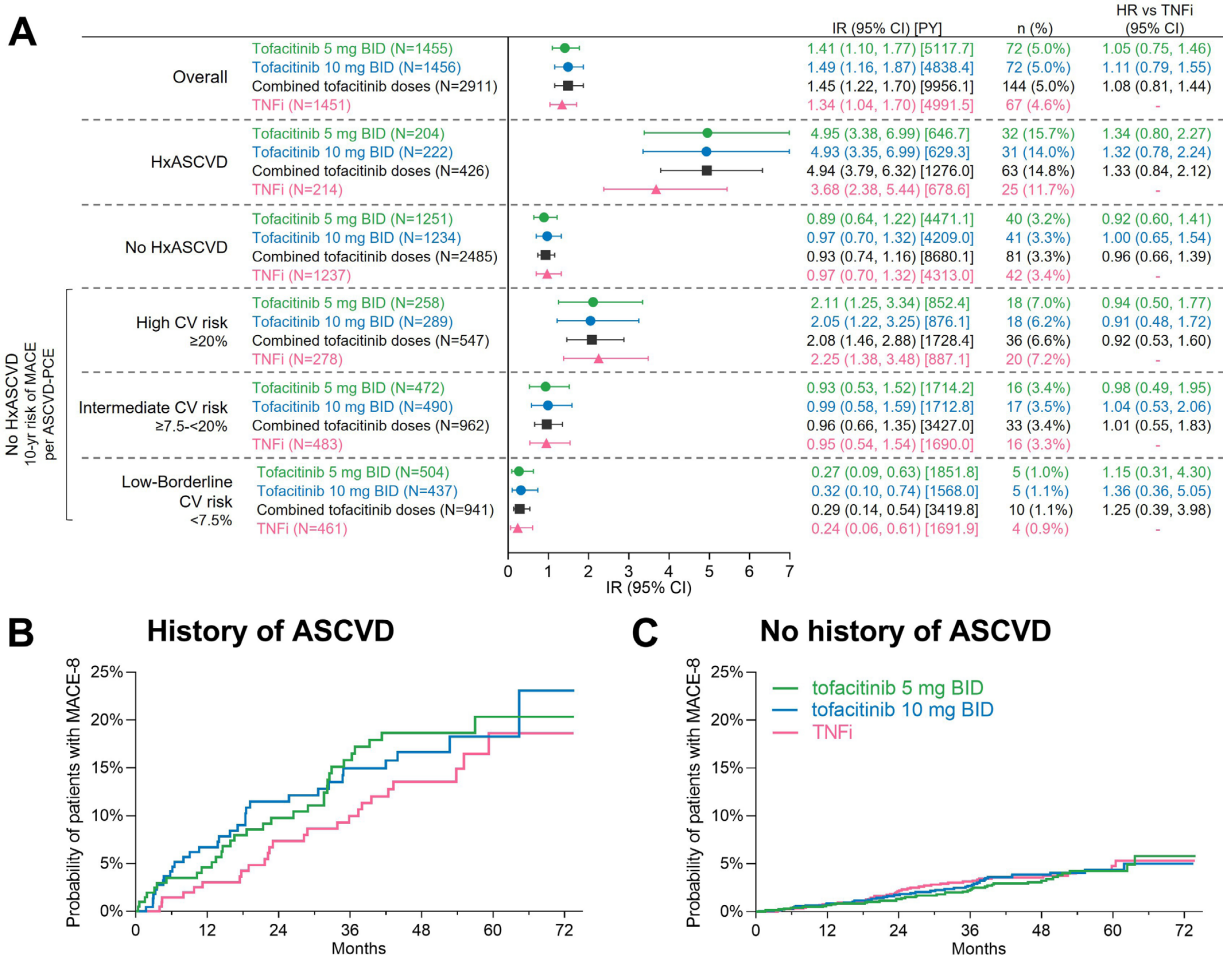
Risk of MACE-8 with tofacitinib versus TNFi by history of ASCVD and by levels of predicted 10-year risk of MACE. ‘MACE-8’ is a composite of all adjudicated CV events (not including VTE) in ORAL Surveillance. (A) IRs express the number of patients with first events per 100 PY. HRs (95% CIs) are from time to first event analyses based on two simple Cox proportional hazard models: one comparing tofacitinib 5 mg two times per day and 10 mg two times per day versus TNFi, and the other comparing combined tofacitinib doses versus TNFi. Because of missing ASCVD-PCE score, four CV events could not be associated with baseline CV risk (n=1 in the tofacitinib 5 mg two times per day group, n=1 in the tofacitinib 10 mg two times per day group, and n=2 in the TNFi group. (B, C) Cumulative probabilities of patients with MACE-8 calculated based on the Kaplan-Meier estimates. ASCVD, atherosclerotic cardiovascular disease; IR, incidence rate; MACE, major adverse cardiovascular events; N, number of evaluable patients; n, number of patients with events; PY, patient-years; TNFi, tumour necrosis factor inhibitor; VTE, venous thromboembolism.

Risk of MACE-8 (ie, all ischaemic CV events plus HF, but not including VTE) appeared higher with tofacitinib versus TNFi in patients with history of ASCVD (figure 5A). In patients with no history of ASCVD, a difference in risk of MACE-8 (figure 5A) was not suggested. Figure 5A (lower half) shows risk of MACE-8 with tofacitinib versus TNFi in patients with no history of ASCVD and high, intermediate or low-borderline predicted 10-year risk of MACE at baseline. Across these risk categories, any difference in risk of MACE-8 between tofacitinib and TNFi was not apparent.

The Kaplan-Meier curve for MACE-8 in patients with history of ASCVD indicated very early separation (as early as 3–6 months) between the tofacitinib and TNFi groups (figure 5B). In patients with no history of ASCVD, there was no separation between any of the treatment groups (figure 5C). The Kaplan-Meier curves also highlight the relatively large difference in absolute risk of MACE-8 in patients with or without history of ASCVD (figure 4B).

The risk of MACE-8 plus VTE in the overall population was increased with tofacitinib 10 mg two times per day versus TNFi (figure 2). This risk difference was observed in patients with a history of ASCVD and patients without a history of ASCVD, including across categories of predicted CV risk (online supplemental figure 2).

### Risk of extended MACE endpoints with tofacitinib versus TNFi by age (≥65 or <65 years) and by geographical region

**Figure 6:**
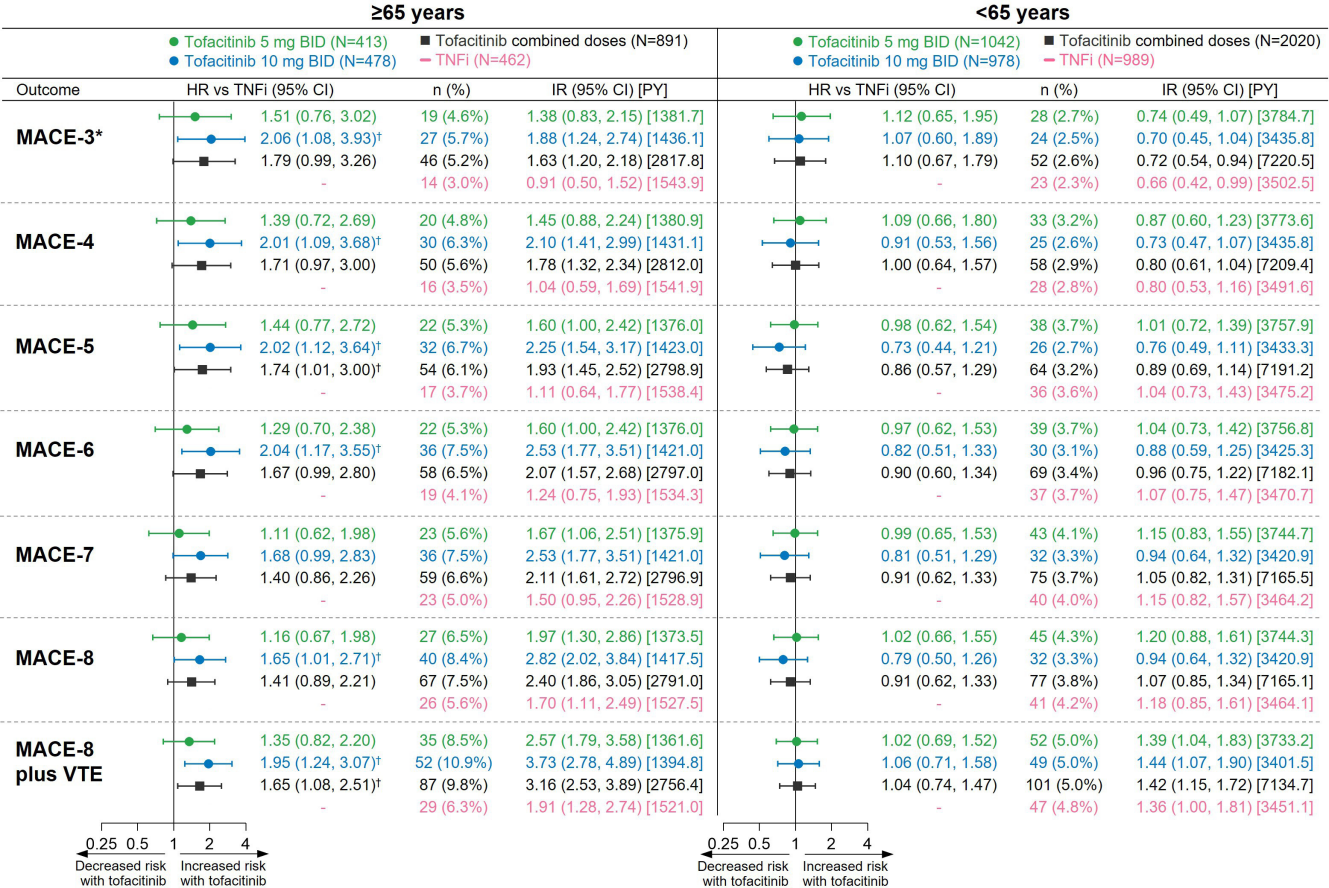
Risk of extended MACE endpoints with tofacitinib versus TNFi in patients ≥65 or <65 years of age. *Results reported in Ytterberg *et al* 10 and included for reference. ^†^HR 95% CI excludes 1. HRs (95% CIs), shown on a logarithmic scale, are from time to first event analyses based on two simple Cox proportional hazard models: one comparing tofacitinib 5 mg two times per day and 10 mg two times per day versus TNFi, and the other comparing combined tofacitinib doses versus TNFi. IRs express the number of patients with first events per 100 PY. IR, incidence rate; MACE, major adverse cardiovascular events; N, number of evaluable patients; n, number of patients with events; PY, patient-years; TNFi, tumour necrosis factor inhibitor; VTE, venous thromboembolism.

A previous subgroup analysis showed that risk of MACE-3 with tofacitinib versus TNFi was more pronounced in patients ≥65 years than in patients <65 years of age.^10^ Across the extended MACE definitions, a similar pattern was found (figure 6). For most of the extended MACE endpoints in patients ≥65 years of age, the 95% CI of the HR for tofacitinib 10 mg two times per day versus TNFi excluded 1. In patients <65 years of age, a difference in risk with tofacitinib versus TNFi was not apparent for any of the extended endpoints, including MACE-8 plus VTE (HRs 0.91–1.04 for the combined tofacitinib doses vs TNFi; figure 6).

Consistent with a previous analysis and likely reflecting overall higher CV risk (online supplemental table 2),^10^ extended MACE endpoints were more frequent among patients in North America than in patients in rest of the world (online supplemental figure 3). However, the relative risk of the extended MACE endpoints with tofacitinib vs TNFi in the geographical regions (online supplemental figure 3) was similar to what was observed in the overall population (figures 1 and 2).

## Discussion

This post hoc analysis shows that with the extension of the coprimary MACE-3 endpoint of ORAL Surveillance to a composite of all ischaemic CV events and hospitalisation for HF as MACE-8, no risk difference was suggested with tofacitinib versus TNFi. The totality of CV risk also including VTE (ie, MACE-8 plus VTE) was higher with tofacitinib 10 mg two times per day versus TNFi but appeared similar with tofacitinib 5 mg two times per day versus TNFi. Risk of MI was increased with tofacitinib versus TNFi, but a difference in risk of other individual CV events with tofacitinib versus TNFi was not suggested. Finally, across extended MACE endpoints (ie, MACE-4 to MACE-8), risk was numerically higher with tofacitinib versus TNFi in patients with a history of ASCVD and higher in patients ≥65 years of age, but similar in patients with no history of ASCVD or patients younger than 65 years.

The analyses expand on primary and secondary analyses of ORAL Surveillance by extending the coprimary endpoint of MACE-3 to composites that include a wider range of CV events and with reporting on additional individual CV outcomes. Extending MACE-3 with additional CV outcomes may inform on potential pathophysiological mechanisms that underpin any between treatments differences. It also means that the risk estimates for the composites were based on more events, providing better statistical precision. Therefore, even though the HRs with tofacitinib versus TNFi for MACE-4 to MACE-8 were similar to that for MACE-3, their 95% CIs were narrower and largely overlapping. MACE-7 included all adjudicated CV outcomes that would be expected to be associated with lipid abnormalities (ie, ischaemic CV events associated with ASCVD),^9^ the observation that prompted ORAL Surveillance.^10^ Notably, a difference in risk of MACE-7 with tofacitinib versus TNFi (HR 1.07 (95% CI 0.79 to 1.44)) was not apparent. Adding outcomes to the MACE-3 composite that may be less influenced by tofacitinib or TNFi could increase the likelihood of shifting the HR for a first event towards neutrality^16–18^ so evaluating risk of the individual CV outcomes is also important. Secondary analyses of ORAL Surveillance indicated that increased risk of MI was an important driver of the difference in the risk of the coprimary endpoint, MACE-3, with tofacitinib versus TNFi^12^ and risk of VTE was increased with tofacitinib 10 mg two times per day versus TNFi.^10^ In this current analysis, apart from risk of MI, a risk difference was not suggested for other individual CV outcomes with tofacitinib versus TNFi. In addition, while risk of MACE-8 appeared similar with tofacitinib 10 mg two times per day versus TNFi, extending the endpoint to include VTE revealed an increase in the totality of CV risk driven by events of VTE.

A history of ASCVD is an important risk factor for future MACE in the general population.^9^ It was, therefore, unsurprising that this was also observed with both tofacitinib and TNFi treatment groups individually. However, this previous history of ASCVD also appeared to be a differentiating risk factor for the increased CV outcomes observed with tofacitinib versus TNFi^12^ seen consistently, across the extended MACE endpoints. A clear separation was seen within 3 months between the tofacitinib and the TNFi treatment arms in patients with a history of ASCVD for MACE-8, the composite of all ischaemic CV events and HF. In contrast, in patients with no history of ASCVD, there was no difference in risk observed with tofacitinib versus TNFi across the extended endpoints. Collectively, the findings by history of ASCVD suggest that the increased relative risk with tofacitinib versus TNFi is dependent on established atherosclerotic disease. Our findings also highlight the absolute risk associated with prevalent ASCVD in patients with RA. A previous analysis of ORAL Surveillance showed that this group did not receive adequate secondary CVD prevention with approximately only 50% of patients with history of ASCVD on statins at baseline.^12^ Age is an important CV risk factor in the general population since atherosclerosis progresses with advancing age.^14^ Age ≥65 years is regarded as a high-risk condition in patients with ASCVD,^14^ and in our analysis, this patient group compared with patients younger than 65 years was two times as likely to have a history of ASCVD. The subgroup analysis presented here on risk of extended MACE endpoints in patients ≥65 or <65 years of age aligns well with a previous analysis on MACE-3. This includes the observation that risk of extended MACE endpoints was increased with tofacitinib 10 mg two times per day versus TNFi in patients ≥65 years of age.

The data presented herein and in previous analyses of ORAL Surveillance indicate that the increased CV risk associated with tofacitinib versus TNFi mainly involves MI and specifically for tofacitinib 10 mg two times per day versus TNFi, VTE.^10 12^ The mechanisms for the increased risk of these events are currently not understood. Although thrombotic events such as MI and VTE share several risk factors,^19^ these outcomes are triggered by distinct pathogenic processes. MI is predominantly a consequence of rupture of a coronary artery plaque,^20^ while VTE is associated with formation of thrombi due to hypercoagulability in the venous compartment.^19^ The finding of increased risk of MI with tofacitinib versus TNFi across the extended MACE, in particular, implies that the treatment difference effect appears to be mainly with acute events and that too of the coronary artery vasculature. This difference in relative risk may conceivably also suggest TNFi as more atheroprotective. Currently, we do not know mechanisms for the increased risk of VTE with tofacitinib 10 mg two times per day versus TNFi.^21^ The risk seems to particularly involve PE,^10^ a finding that has been replicated in a study of Janus kinase inhibitors versus other forms of treatment for RA in a real-world healthcare setting.^22^ Notably, a recent post hoc analysis of ORAL Surveillance suggested that the higher risk of VTE with tofacitinib versus TNFi was restricted to patients ≥65 years of age and/or with a history of long-term current/past smoking.^23^ Future studies will provide more clarity on specific patient characteristics associated with increased risk of MI and VTE with tofacitinib vs TNFi.

A key limitation of this analysis is its exploratory nature such that the results should be interpreted with caution and as hypothesis generating. Some of the endpoints require clinical judgement (eg, coronary revascularisation), which may, despite adjudication of these CV events, introduce bias.^24^ Finally, for some of the individual CV outcomes, the number of events were low, and the related risk estimates need to be interpreted carefully. CV risk estimated with ASCVD-PCE was used to categorise patients in some analyses. ASCVD-PCE was validated with data from a US population.^25^ The application of the calculator on patients from racial/ethnic groups or from geographical regions where it was not explicitly validated could have resulted in inadvertently low or high predicted CV risk.

In conclusion, in this post hoc analysis of ORAL Surveillance, the risk of extended ischaemic events and/or hospitalisation for HF did not appear different with tofacitinib 5 mg two times per day and 10 mg two times per day versus TNFi. Risk was increased in all treatment groups in those with a history of ASCVD or age ≥65 years with the greater risk with tofacitinib versus TNFi also in these groups. Increase in VTE events with tofacitinib 10 mg two times per day versus TNFi underpinned the increased totality of CV risk, which appeared similar with tofacitinib 5 mg two times per day versus TNFi. The increased risk of mainly MI and VTE with tofacitinib versus TNFi warrants further mechanistic evaluation.

## Data Availability

Data are available upon reasonable request. Upon request, and subject to review, Pfizer will provide the data that support the findings of this study. Subject to certain criteria, conditions, and exceptions, Pfizer may also provide access to the related individual de-identified participant data.See https://www.pfizer.com/science/clinical-trials/trial-data-and-results for more information.
