# Supplementary material for: Risk of extended major adverse cardiovascular event endpoints with tofacitinib versus TNF inhibitors in patients with rheumatoid arthritis: a post hoc analysis of a phase 3b/4 randomised safety study
Source: RMD Open. 2024 Apr 12;10(2):e003912. doi: 10.1136/rmdopen-2023-003912 (PMC11029242; doi:10.1136/rmdopen-2023-003912)
Supplement: Supplementary data [file rmdopen-2023-003912supp001.pdf]

BMJ Publishing Group Limited (BMJ) disclaims all liability and responsibility arising from any reliance placed on this supplemental material which has been supplied by the author(s)

SUPPLEMENTAL MATERIAL

Online supplemental table S1. Key demographic and baseline disease characteristics of patients ≥65 or <65 years at baseline of ORAL Surveillance. 2

Online supplemental table S2. Key demographic and baseline disease characteristics of patients in ORAL Surveillance by geographical regions. 3

Online supplemental table S3. Characteristics of events of MI in patients treated with tofacitinib or TNFi. 4

Supplemental figure S1. Risk of total (i.e., first and subsequent) ischemic CV events (MACE-7) with tofacitinib vs TNFi. 5

Supplemental figure S2. Risk of MACE-8 plus VTE with tofacitinib vs TNFi in patients with or without history of ASCVD. 6

Supplemental figure S3. Risk of extended MACE endpoints with tofacitinib vs TNFi in patients by geographical region. 7

References 8

Buch MH, *et al. RMD Open* 2024; 10:e003912. doi: 10.1136/rmdopen-2023-003912

Online supplemental table S1. Key demographic and baseline disease characteristics of patients ≥65 or <65 years at baseline of ORAL Surveillance.

	≥65 years			<65 years		
	**Tofacitinib 5 mg BID (N=413)**	**Tofacitinib 10 mg BID (N=478)**	**TNFi (N=462)**	**Tofacitinib 5 mg BID (N=1,042)**	**Tofacitinib 10 mg BID (N=978)**	**TNFi (N=989)**
Age (years), mean (SD)	69.5 (4.0)	69.7 (4.2)	69.0 (4.7)	57.3 (4.0)	57.3 (4.0)	57.2 (4.2)
Female sex, n (%)	333 (80.6)	364 (76.2)	361 (78.1)	836 (80.2)	760 (77.7)	756 (76.4)
Race*, n (%)						
White	339 (82.1)	398 (83.3)	361 (78.1)	789 (75.7)	728 (74.4)	738 (74.6)
Black	18 (4.4)	21 (4.4)	32 (6.9)	45 (4.3)	44 (4.5)	51 (5.2)
Asian	10 (2.4)	14 (2.9)	11 (2.4)	55 (5.3)	42 (4.3)	44 (4.4)
Other	46 (11.1)	45 (9.4)	58 (12.6)	153 (14.7)	164 (16.8)	156 (15.8)
Smoking status, n (%)						
Current smoker	93 (22.5)	93 (19.5)	74 (16.0)	318 (30.5)	309 (31.6)	279 (28.2)
Past smoker	108 (26.2)	126 (26.4)	141 (30.5)	201 (19.3)	176 (18.0)	185 (18.7)
Never smoked	212 (51.3)	259 (54.2)	247 (53.5)	523 (50.2)	493 (50.4)	525 (53.1)
Smoking duration, years (SD)[†]						
Current smokers	39.0 (15.2)	39.1 (15.2)	40.5 (12.8)	30.2 (11.7)	31.4 (11.7)	29.7 (12.4)
Past smokers	43.6 (13.5)	42.3 (14.5)	46.3 (11.7)	32.6 (11.0)	35.0 (10.8)	35.0 (10.2)
Time since smoking cessation (past smokers), years (SD)	17.9 (13.8)	16.8 (12.2)	22.4 (13.5)	12.8 (11.0)	15.9 (11.8)	16.1 (12.1)
BMI (kg/m^2) ≥30[‡], n (%)	169 (40.9)	201 (42.1)	181 (39.2)	437 (41.9)	393 (40.2)	436 (44.1)
History of diabetes mellitus, n (%)	81 (19.6)	110 (23.0)	89 (19.3)	162 (15.5)	151 (15.4)	166 (16.8)
History of hypertension, n (%)	309 (74.8)	356 (74.5)	352 (76.2)	646 (62.0)	598 (61.1)	617 (62.4)
History of hyperlipidemia, n (%)	186 (45.0)	215 (45.0)	188 (40.7)	339 (32.5)	303 (31.0)	303 (30.6)
Baseline statin[§], n (%)	139 (33.7)	161 (33.7)	138 (29.9)	210 (20.2)	189 (19.3)	183 (18.5)
Baseline aspirin[§], n (%)	90 (21.8)	118 (24.7)	104 (22.5)	122 (11.7)	113 (11.6)	120 (12.1)
History of ASCVD, n (%)	84 (20.3)	109 (22.8)	113 (24.5)	120 (11.5)	113 (11.6)	101 (10.2)
10-year risk of MACE,[**] n (%)						
High (≥20%)	176 (42.6)	207 (43.3)	200 (43.3)	82 (7.9)	82 (8.4)	78 (7.9)
Intermediate (≥7.5–<20%)	135 (32.7)	142 (29.7)	132 (28.6)	337 (32.3)	348 (35.6)	351 (35.5)
Borderline (≥5–<7.5%)	8 (1.9)	8 (1.7)	6 (1.3)	190 (18.2)	161 (16.5)	147 (14.9)
Low (<5%)	1 (0.2)	2 (0.4)	3 (0.6)	305 (29.3)	266 (27.2)	305 (30.8)

*Race was reported by the patient. [†]Information on smoking duration was missing on eight patients (two current and six past smokers) treated with tofacitinib and five patients (one current and four past smokers) treated with TNFi. In the past smokers, data were also missing on time since smoking cessation. [‡]Across treatment groups, data were missing in 17 patients (0.4%). [§]Based on day 1 of treatment with tofacitinib or TNFi in ORAL Surveillance. [**]In patients with no history of ASCVD, a 10-year risk of MACE was calculated with the ASCVD-PCE calculator,[1] and a 1.5 multiplier was applied for RA, as recommended by EULAR.[2] Percentages are calculated based on the total N.

ASCVD, atherosclerotic cardiovascular disease; ASCVD-PCE, ASCVD-Pooled Cohort Equations; EULAR, European Alliance of Associations for Rheumatology; N, number of evaluable patients; n, number of patients with a characteristic; SD, standard deviation.

Online supplemental table S2. Key demographic and baseline disease characteristics of patients in ORAL Surveillance by geographical regions.

	North America (US, Puerto Rico, and Canada [adalimumab])			Rest of the world (etanercept)		
	Tofacitinib 5 mg BID (N=402)	Tofacitinib 10 mg BID (N=409)	TNFi (N=432)	Tofacitinib 5 mg BID (N=1,053)	Tofacitinib 10 mg BID (N=1,047)	TNFi (N=1019)
Age (years), mean (SD)	62.2 (7.2)	62.8 (7.4)	63.3 (8.1)	60.2 (6.6)	60.9 (6.9)	60.5 (7.0)
≥65 years, n (%)	145 (36.1)	171 (41.8)	182 (42.1)	268 (25.5)	307 (29.3)	280 (27.5)
Female sex, n (%)	305 (75.9)	300 (73.3)	303 (70.1)	864 (82.1)	824 (78.7)	814 (79.9)
Race*, n (%)						
White	338 (84.1)	352 (86.1)	363 (84.0)	790 (75.0)	774 (73.9)	736 (72.2)
Black	46 (11.4)	42 (10.3)	61 (14.1)	17 (1.6)	23 (2.2)	22 (2.2)
Asian	4 (1.0)	8 (2.0)	3 (0.7)	61 (5.8)	48 (4.6)	52 (5.1)
Other	14 (3.5)	7 (1.7)	5 (1.2)	185 (17.6)	202 (19.3)	209 (20.5)
Smoking status, n (%)						
Current smoker	106 (26.4)	121 (29.6)	95 (22.0)	305 (29.0)	281 (26.8)	258 (25.3)
Past smoker	117 (29.1)	117 (28.6)	150 (34.7)	192 (18.2)	185 (17.7)	176 (17.3)
Never smoked	179 (44.5)	171 (41.8)	187 (43.3)	556 (52.8)	581 (55.5)	585 (57.4)
Smoking duration, years (SD)†						
Current smokers	37.8 (11.2)	38.8 (12.4)	37.7 (12.1)	30.2 (13.2)	30.7 (12.5)	29.8 (13.0)
Past smokers	39.7 (12.4)	42.2 (12.9)	43.2 (11.7)	34.5 (13.1)	35.5 (12.4)	37.0 (12.0)
Time since smoking cessation (past smokers), years (SD)	16.2 (13.9)	16.7 (11.8)	16.4 (12.9)	13.6 (11.0)	16.0 (12.1)	16.8 (12.3)
BMI (kg/m^2) ≥30‡, n (%)	209 (52.0)	237 (57.9)	260 (60.2)	397 (37.7)	357 (34.1)	357 (30.0)
History of diabetes mellitus, n (%)	102 (25.4)	100 (24.4)	109 (25.2)	141 (13.4)	161 (15.4)	146 (14.3)
History of hypertension, n (%)	280 (69.7)	297 (72.6)	309 (71.5)	675 (64.1)	657 (62.8)	660 (64.8)
History of hyperlipidemia, n (%)	198 (49.3)	205 (50.1)	205 (47.5)	327 (31.1)	313 (29.9)	286 (28.1)
Baseline statin§, n (%)	135 (33.6)	151 (36.9)	139 (32.2)	214 (20.3)	199 (19.0)	182 (17.9)
Baseline aspirin§, n (%)	110 (27.4)	118 (28.9)	121 (28.0)	102 (9.7)	113 (10.8)	103 (10.1)
History of ASCVD, n (%)	72 (17.9)	81 (19.8)	94 (21.8)	132 (12.5)	141 (13.5)	120 (11.8)
10-year risk of MACE,** n (%)						
High (≥20%)	89 (22.1)	103 (25.2)	109 (25.2)	169 (12.5)	186 (13.5)	169 (11.8)
Intermediate (≥7.5–<20%)	127 (31.6)	135 (33.0)	131 (30.3)	345 (32.8)	355 (33.9)	352 (34.5)
Borderline (≥5–<7.5%)	48 (11.9)	30 (7.3)	30 (6.9)	150 (14.2)	139 (13.3)	123 (12.1)
Low (<5%)	59 (14.7)	52 (12.7)	64 (14.8)	247 (23.5)	216 (20.6)	244 (23.9)

*Race was reported by the patient. †Information on smoking duration was missing on eight patients (two current and six past smokers) treated with tofacitinib and five patients (one current and four past smokers) treated with TNFi. In the past smokers, data were also missing on time since smoking cessation. ‡Across treatment groups, data were missing in 17 patients (0.4%). §Based on day 1 of treatment with tofacitinib or TNFi in ORAL Surveillance. **In patients with no history of ASCVD, a 10-year risk of MACE was calculated with the ASCVD-PCE calculator,[1] and a 1.5 multiplier was applied for RA, as recommended by EULAR.[2] Percentages are calculated based on the total N.

ASCVD, atherosclerotic cardiovascular disease; ASCVD-PCE, ASCVD-Pooled Cohort Equations; EULAR, European Alliance of Associations for Rheumatology; N, number of evaluable patients; n, number of patients with a characteristic; SD, standard deviation; US, Unites States.

Online supplemental table S3. Characteristics of events of MI in patients treated with tofacitinib or TNFi.

	Tofacitinib 5 mg BID (N=1455)	Tofacitinib 10 mg BID (N=1456)	TNFi (N=1451)
Total MI*, n (%)	19 (1.3%)	19 (1.3%)	11 (0.8%)
Fatal MI*, n (%)	0 (0.0%)	3 (0.2%)	3 (0.2%)
Non-fatal MI*, n (%)	19 (1.3%)	16 (1.1%)	8 (0.6%)
Classification of MI, n (%)[†]			
STEMI	10 (52.6%)	4 (21.1%)	5 (45.5%)
NSTEMI	7 (36.8%)	11 (57.9%)	4 (36.4%)
Undetermined	2 (10.5%)	4 (21.1%)	2 (18.2%)

*Previously reported and included for reference.[3] [†]1 fatal MI with tofacitinib 10 mg BID and 1 fatal MI with TNFi was reported as STEMI, the other fatal events were not possible to classify.

MI, myocardial infarction; NSTEMI, non-ST elevation MI; STEMI, ST elevation MI

Buch MH, *et al. RMD Open* 2024; 10:e003912. doi: 10.1136/rmdopen-2023-003912

Supplemental figure S1. Risk of total (i.e., first and subsequent) ischemic CV events (MACE-7) with tofacitinib vs TNFi.

		HR vs TNFi (95% CI)	Total nr of events	Pts with ≥1 event (%)	Pts with 1 event (%)	Pts with 2 events (%)
**Overall population**	Tofacitinib 5 mg BID (N=1455)	0.99 (0.71, 1.38)	71	66 (4.5%)	61 (4.2%)	5 (0.3%)
	Tofacitinib 10 mg BID (N=1456)	1.09 (0.79, 1.52)	74	68 (4.7%)	63 (4.3%)	4 (0.3%)
	Combined tofacitinib doses (N=2911)	1.04 (0.78, 1.38)	145	134 (4.6%)	124 (4.3%)	9 (0.3%)
	TNFi (N=1451)		70	63 (4.3%)	57 (3.9%)	5 (0.3%)
**HxASCVD**	Tofacitinib 5 mg BID (N=204)	1.18 (0.70, 1.99)	30	27 (13.2%)	24 (11.8%)	3 (1.5%)
	Tofacitinib 10 mg BID (N=222)	1.22 (0.72, 2.06)	30	29 (13.1%)	28 (12.6%)	1 (0.5%)
	Combined tofacitinib doses (N=426)	1.20 (0.76, 1.90)	60	56 (13.2%)	52 (12.2%)	4 (0.9%)
	TNFi (N=214)		26	22 (10.2%)	19 (8.9%)	2 (0.9%)
**No HxASCVD**	Tofacitinib 5 mg BID (N=1251)	0.90 (0.59, 1.38)	41	39 (3.1%)	37 (3.0%)	2 (0.2%)
	Tofacitinib 10 mg BID (N=1234)	1.03 (0.68, 1.56)	44	39 (3.2%)	35 (2.8%)	3 (0.2%)
	Combined tofacitinib doses (N=2485)	0.96 (0.67, 1.39)	85	78 (3.1%)	72 (2.9%)	5 (0.2%)
	TNFi (N=1237)		44	41 (3.3%)	38 (3.1%)	3 (0.2%)

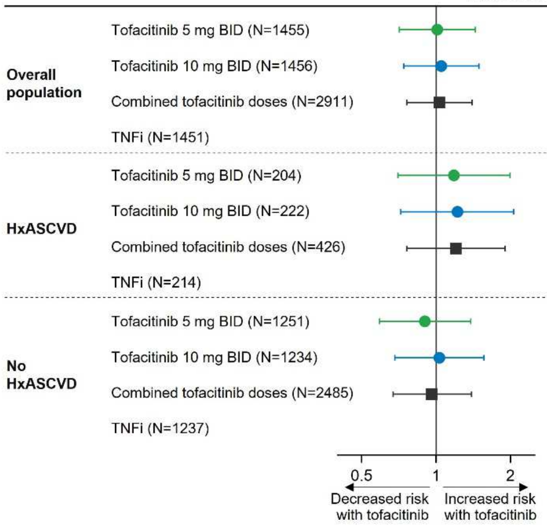

Total ischemic CV events include first and recurrent adjudicated events defined in the MACE-7 composite (CV death, myocardial infarction, stroke, hospitalization for unstable angina, coronary revascularization, transient ischemic attack, and peripheral vascular disease). Events that occurred on the same day were considered as a single event.

HRs (95% CIs) were determined using Andersen-Gill model.

ASCVD, atherosclerotic cardiovascular disease; BID, twice daily; CI, confidence interval; CV, cardiovascular; HR, hazard ratio; Hx, history of; IR, incidence rate; MACE, major adverse cardiovascular events; n, number of patients with events; N, number of evaluable patients; Pts, patients; TNFi, tumour necrosis factor inhibitor; VTE, venous thromboembolism.

Buch MH, *et al. RMD Open* 2024; 10:e003912. doi: 10.1136/rmdopen-2023-003912

Supplemental figure S2. Risk of MACE-8 plus VTE with tofacitinib vs TNFi in patients with or without history of ASCVD.

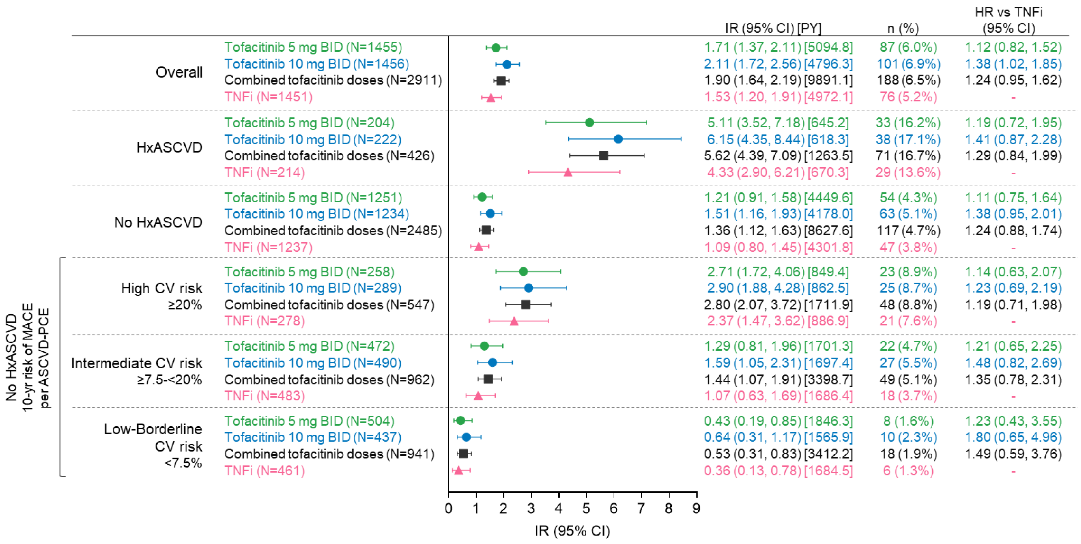

IRs express the number of patients with first events per 100 PY. HRs (95% CIs) are from time to first event analyses based on two simple Cox proportional hazard models: one comparing tofacitinib 5 mg BID and 10 mg BID vs TNFi, and the other comparing combined tofacitinib doses vs TNFi. Because of missing ASCVD-PCE score, four CV events could not be associated with baseline CV risk (n=1 in the tofacitinib 5 mg BID group, n=1 in the tofacitinib 10 mg BID group, and n=2 in the TNFi group.

ASCVD, atherosclerotic cardiovascular disease; BID, twice daily; CI, confidence interval; CV, cardiovascular; HR, hazard ratio; IR, incidence rate; MACE, major adverse cardiovascular events; n, number of patients with events; N, number of evaluable patients; PCE, pooled cohort equations; PY, patient-years; TNFi, tumour necrosis factor inhibitor; VTE, venous thromboembolism.

Buch MH, et al. RMD Open 2024; 10:e003912. doi: 10.1136/rmdopen-2023-003912

Supplemental figure S3. Risk of extended MACE endpoints with tofacitinib vs TNFi in patients by geographical region.

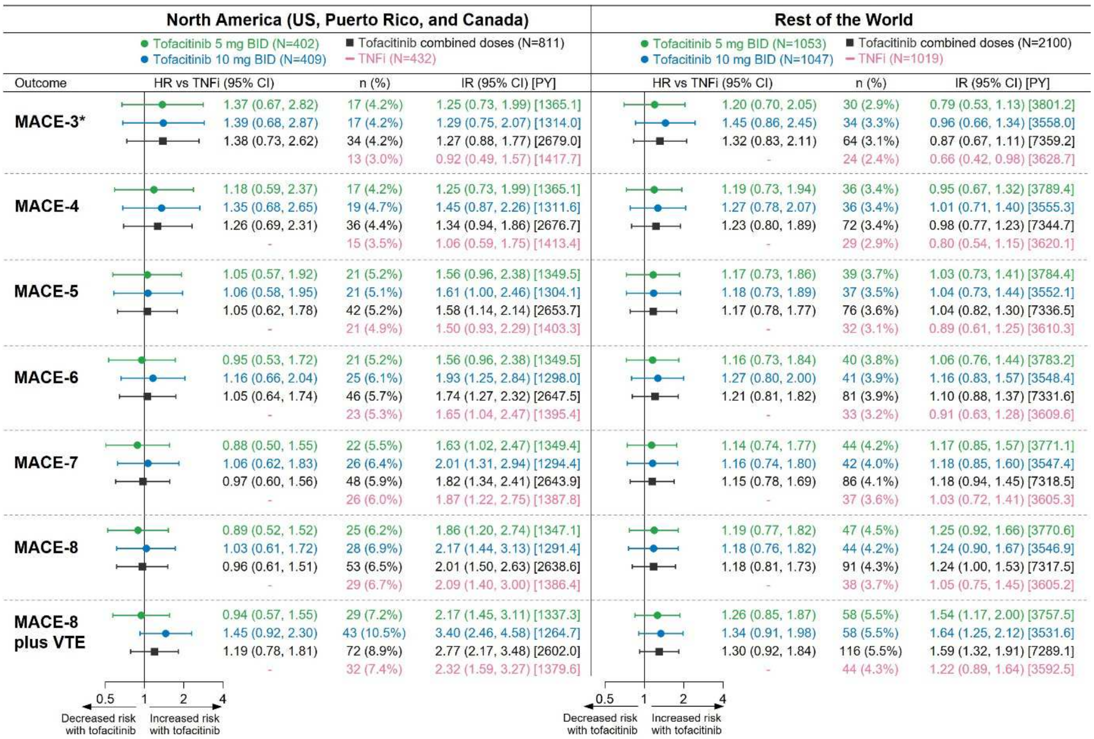

*Results reported in Ytterberg et al.[4] and included for reference. HRs (95% CIs), shown on a logarithmic scale, are from time to first event analyses based on two simple Cox proportional hazard models: one comparing tofacitinib 5 mg BID and 10 mg BID vs TNFi, and the other comparing combined tofacitinib doses vs TNFi. IRs express the number of patients with first events per 100 PY.

BID, twice daily; CI, confidence interval; HR, hazard ratio; IR, incidence rate; MACE, major adverse cardiovascular events; N, number of evaluable patients; n, number of patients with events; PY, patient-years; TNFi, tumour necrosis factor inhibitor; US, United States.

Buch MH, *et al. RMD Open* 2024; 10:e003912. doi: 10.1136/rmdopen-2023-003912

References

1. Grundy SM, Stone NJ, Bailey AL, et al. 2018 AHA/ACC/AACVPR/AAPA/ABC/ACPM/ADA/AGS/APhA/ASPC/NLA/PCNA Guideline on the Management of Blood Cholesterol: A Report of the American College of Cardiology/American Heart Association Task Force on Clinical Practice Guidelines. *J Am Coll Cardiol* 2019;73:e285-e350.
2. Agca R, Heslinga SC, Rollefstad S, et al. EULAR recommendations for cardiovascular disease risk management in patients with rheumatoid arthritis and other forms of inflammatory joint disorders: 2015/2016 update. *Ann Rheum Dis* 2017;76:17-28.
3. Charles-Schoeman C, Buch MH, Dougados M, et al. Risk of major adverse cardiovascular events with tofacitinib versus tumour necrosis factor inhibitors in patients with rheumatoid arthritis with or without a history of atherosclerotic cardiovascular disease: a post hoc analysis from ORAL Surveillance. *Ann Rheum Dis* 2023;82:119-29.
4. Ytterberg SR, Bhatt DL, Mikuls TR, et al. Cardiovascular and Cancer Risk with Tofacitinib in Rheumatoid Arthritis. *N Engl J Med* 2022;386:316-26.
